# Fabrication of composite transparent conductive electrodes based on silver nanowires

**DOI:** 10.1038/s41598-024-53286-8

**Published:** 2024-02-06

**Authors:** Amal Elsokary, Moataz Soliman, Fuad Abulfotuh, Shaker Ebrahim, Torfeh Sadat-Shafai, Marwa Karim

**Affiliations:** 1 Department of Materials Science, Institute of Graduate Studies and Research, Alexandria University, P.O. Box 21526, Alexandria, Egypt; 2 Department of Engineering, School of Digital, Technologies and Arts, Staffordshire University, Manchester, UK; 3 Physics Department, Faculty of Science, Alexandria University, Moharram Bek, P.O. Box 21511, Alexandria, Egypt

**Keywords:** Energy science and technology, Materials science, Nanoscience and technology

## Abstract

Composite transparent conductive electrodes (C-TCEs) have recently been produced using low-cost techniques to keep up with the boom in the fabrication and development of optoelectronic devices. In this article, silver nanowires (AgNWs) were successfully synthesized by a simple hydrothermal method using different molecular weights M_W_s of poly (N-vinylpyrrolidone) (PVP). Graphene oxide (GO) was prepared using the modified Hummers’ method and a reduction step was held on GO films to produce reduced GO (rGO). C-TCEs were fabricated by over-coating the AgNWs electrodes with rGO, or poly(3,4-ethylenedioxythiophene) polystyrene sulfonate to improve the roughness, surface energy, and sheet resistance. The influence of using lower and higher M_W_s of PVP on the yield, shape, and size of AgNWs was investigated. The results showed that using lower M_W_ of PVP had a great effect on the yield, morphology, and aspect ratio of AgNWs with diameter of 46 nm and average length 12 µm. The optical, morphological, topographical, and electrical properties of TCEs were studied. AgNWs/rGO composite electrode provided the lowest surface roughness and surface energy of 250 nm and 47.95 mN/m, respectively, with a relatively high transparency of 78.2% at 550 nm light wavelength, and a low sheet resistance of 27 Ω/□.

## Introduction

The rapid development of modern flexible optoelectronic devices such as solar cells, photonic skins, displays, lighting, supercapacitors, and smart windows has prompted researchers and scientists worldwide to design and develop multifunctional, flexible TCE materials^1^. The characteristics required for achieving high-performance TCEs are high optical transmittance, low sheet resistance, low surface roughness, suitable surface work function, high temperature resistance, and low fabrication cost^2^. ITO electrode is commonly used in electronic devices because of its high transmittance of over 80% in the whole visible wavelength region and a particularly low sheet resistance of ≈ 10 Ω/□. On the other hand, the scarcity of indium, the difficulty and expense of fabricating ITO limit its use as a thin film of sufficient quality and brittle nature with a rapidly increased demand for flexible electronic devices^3,4^. Alternative TCEs can be categorized into three primary groups, i.e., carbon based TCEs (graphene, carbon nanotubes (CNTs) and conducting polymers), metal based TCEs (nanostructured films, metal nanowires (MNWs) and metal mesh) and hybrid structured TCEs (a combination of conducting polymers, carbon and metal based TCEs)^5–7^.

AgNWs electrodes are promising candidates as the primary material to replace ITO due to their solubility in different solvents, and high conductivity along with their enhanced properties in terms of light-scattering, plasmonic effects, and transmittance in the near infrared region^8–10^. The most widely used techniques reported for preparation of AgNWs are the polyol method, hydrothermal, and solvothermal synthesis. Hydrothermal is considered a simple and fast way to synthesize AgNWs in one pot using silver nitrate (AgNO_3_) as a silver precursor, glucose as a reducing agent, and PVP as a capping agent to maintain the growth of wires^11^. The literature results indicate that PVP with different molecular weights plays a pivotal role in controlling the shape of the Ag nanostructure due to PVP adsorption on different crystal facets thus affecting the growth rate of AgNWs^12^.

To improve electrical conductivity, air stability, flexibility, surface roughness and often cost effectiveness, composite TCEs have been developed^12–14^. Therefore, the creation of nanocomposite TCEs by coating nanowires with a fully covered protective layer with fewer cracks, such as conductive polymers, and other carbon-based electrodes (carbon nanotubes and graphene) has attracted interest^13–16^.

In this study, a modified hydrothermal method with different PVP molecular weights to assist in the synthesis of high yields and high aspect ratios of AgNWs is adopted. GO is prepared using a modified Hummers’ method followed by thermal and chemical reduction steps to produce rGO conductive films. C-TCEs are fabricated by integrating AgNWs with rGO or PEDOT:PSS materials. Consequently, the optical, structural, and morphological properties of the prepared materials were investigated using ultraviolet–visible (UV–Vis) analysis, Fourier transform infrared spectroscopy (FTIR), Raman spectroscopy, X-ray diffraction (XRD), and high-resolution transmission electron microscopy (HRTEM). Furthermore, novel comparative study of the aspect ratio, roughness, and surface energy between AgNWs, AgNWs/rGO, and AgNWs/PEDOT:PSS are conducted using scanning electron microscopy (SEM), atomic force microscopy (AFM), and contact angle goniometry. Finally, the effect of different architectures of composite TCEs on the transmittance and sheet resistance are plotted using UV–Vis spectroscopy, and a four-point probe.

## Materials and methods

### Materials

Silver nitrate, PVP with Mw 1300,000, PEDOT:PSS 3.0–4.0% in H_2_O, high-conductivity grade, Hellmanex III, and acetone 99.5% were all purchased from Sigma‒Aldrich. PVP with Mw 40,000 was received from LOBA Chemie and D^+^ glucose was obtained from BDH Chemical. Diiodomethane, sulfuric acid 98% and hydrochloric acid 37% were obtained from MERCK. Ethanol 99.8% was purchased from Honeywell. Graphite powder from Bay Carbon, potassium permanganate 99.5% from ACROS ORGANICS, and sodium nitrate 99.5% from ALPHA were all bought. Sodium chloride from PIOCHEM, hydrogen peroxide 50% from ISO-CHEM, and hydrazine monohydrate from Oxford Laboratory were purchased.

### Synthesis of AgNWs

**Figure 1:**
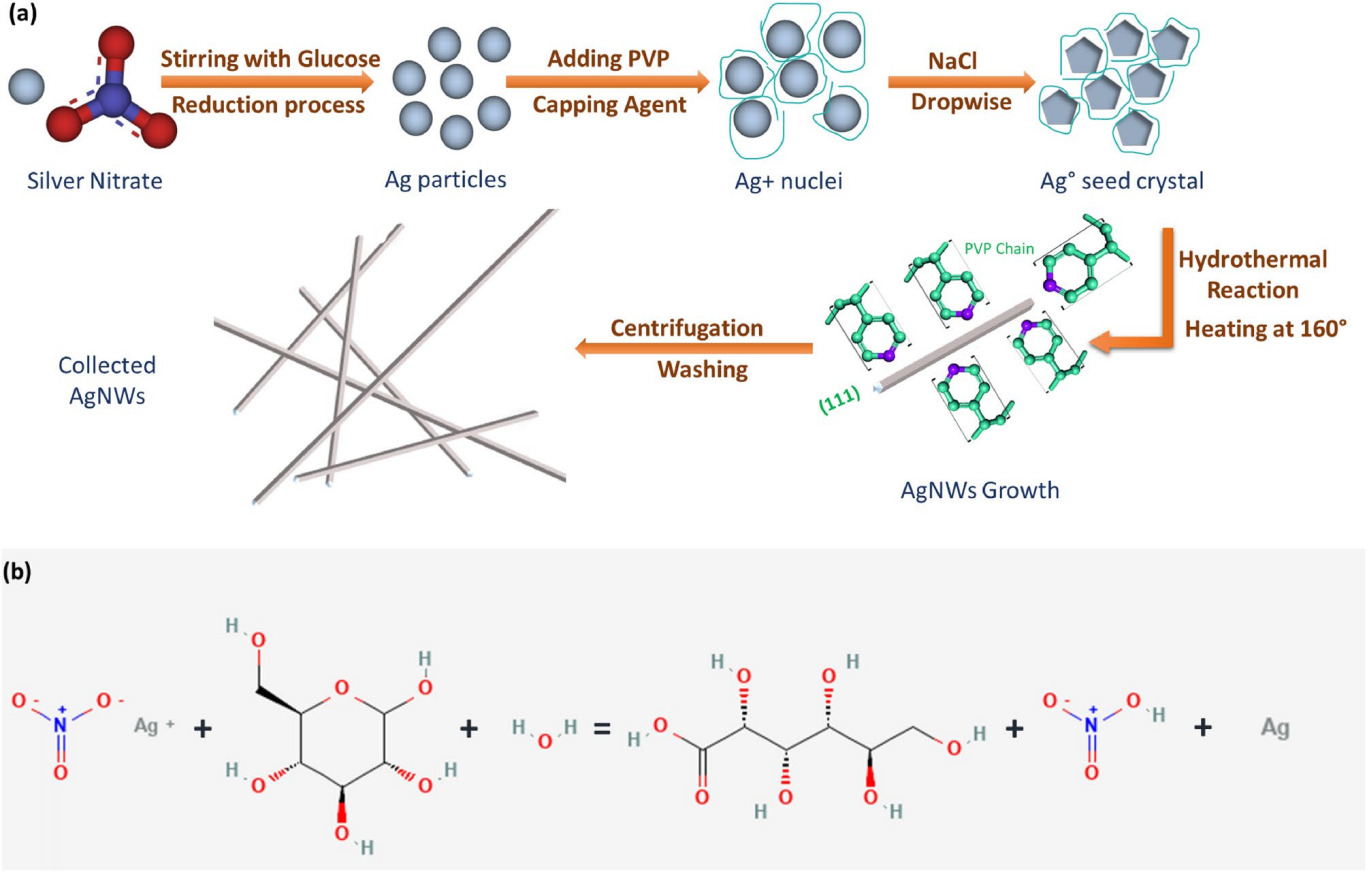
(**a**) A schematic representation demonstrates the step-by-step AgNWs synthesis via the hydrothermal technique, and (**b**) a schematic diagram depicts the reduction process of AgNO_3_ by glucose during the AgNWs synthesis.

Long AgNWs were synthesized by a hydrothermal route. A summary of the AgNWs synthesis procedure is shown in Fig. 1. First, separate solutions of silver nitrate (102 mg, 30 mL), D^+^ glucose (240 mg, 10 mL), sodium chloride (70 mg, 30 mL) and PVP (2 g, 10 mL) were dissolved in deionized water at room temperature except PVP solution was stirred at 65 ℃. In each synthesis, PVP solutions with different M_W_s, PVP_1_ of 40,000 and PVP_2_ of 1300,000, were prepared systemically. After 10 min (mins) of continuous stirring, glucose solution was added to silver nitrate solution to assist in the reduction process. Then, PVP solution was added to the above mixture and stirred for 20 min to obtain a blurred mixture. Grey solution was grown by adding NaCl dropwise to the blurred mixture with continuous stirring for 1 h (h) as a nucleating agent of pentagonal structure seeds of Ag. The obtained turbid mixture was poured into a 100 mL Teflon-lined stainless-steel autoclave and heated in an oven at 160 °C for 22 h. Furthermore, AgNWs in the form of a fluffy gray and white precipitate were formed and gradually cooled to room temperature. Then, the final product was collected by centrifugation at a speed of 3000 rpm for 10 min, and the AgNWs were washed thrice with distilled water and ethanol^11^. Finally, the AgNWs were preserved in ethanol to prevent AgNWs from oxidation and for further use.

### Synthesis of GO

The modified Hummers’ method was utilized to synthesize GO from natural graphite (2 g) and (8 g) of sodium nitrate by adding 50 mL of 98% sulfuric acid^17^. This mixture was stirred for 20 min in an ice-water bath and then, potassium permanganate (12 g) was gradually added over a period of 50 min with a continuous stirring for another 2 h. Afterwards, the above solution was left to reach room temperature to produce a thick slurry. Then, DI water (160 mL) was added slowly, and the solution temperature was raised to 98 °C. Hydrogen peroxide 50% (30 mL) was added to the above suspension after cooling to room temperature to form GO. Finally, the GO solution was washed twice with 300 ml of 5% HCl and DI water three times. After that, GO powder was obtained by filtration and drying in an oven at 60 °C. GO solution in deionized water with concentration (1:1 mg/mL) is obtained to form GO films for further treatment.

Chemical and thermal treatment of GO films by hydrazine vapor was carried out to reduce GO films to rGO. First, GO film inside a glass petri dish containing 1 mL of hydrazine monohydrate 60% was heated at 150 °C for 1 h in a muffle furnace under the flow of nitrogen gas. The color of the films changed from matte brown to metallic gray, indicating a reduction process and formation of rGO layer which can be easily scratched and characterized.

### Fabrication of TCEs

Glass substrates were sequentially cleaned with hellmanex III, DI water, acetone, and isopropanol. Additionally, Clean glass substrates were treated using nitrogen plasma by (Edwards E3064) at high vacuum (2 × 10^–4^ mbar). The glow discharge valve was opened, and pure nitrogen gas was allowed to fill the chamber until the pressure reached 10^–2^ mbar. By applying high tension at 20 mA, a purple color of nitrogen ionization appeared, and the glass samples were left under plasma treatment for 10 min. Transparent conductive films of AgNWs were deposited on glass by a spin coater at 2000 rpm for 30 s. To protect and improve morphological and electrical properties of AgNWs mesh, composite electrodes of AgNWs/rGO or AgNWs/PEDOT:PSS were fabricated. GO and PEDOT:PSS were spin coated on AgNWs films at 2000 and 4000 rpm for 30 s, respectively. Furthermore, AgNWs/PEDOT:PSS electrodes were dried at 50 °C for 1 min and a reduction step for GO is needed to enhance the conductivity and get AgNWs/rGO composite electrodes.

### Characterization techniques and measurements

The absorption and transmittance spectra of AgNWs, GO, rGO and the fabricated electrodes were investigated using an UV–Vis spectrophotometer (Evolution 300 double beam scanning spectrophotometer, Thermo Scientific, USA). FTIR spectra of the prepared materials were obtained using (a spectrum BX 11 spectrometer FTIR LX 18-5255 Perkin Elmer). Raman spectra confirm the formation and reduction of GO (Senterra Raman, Bruker Corporation, USA, λ (532 nm)). X-ray diffraction patterns were obtained for the prepared films using (XRD, D2 PHASER, Bruker) and the X-ray source was a Cu target generated at 30 kV and 40 mA. The contact angles of a drop of water and diiodomethane on AgNWs, AgNWs/rGO, and AgNWs/PEDOT:PSS electrodes were measured before and after treatment using a contact angle goniometer (Ramé-hart CA instrument, model 190-F2). High-resolution transmission electron microscopy (JEOL JEM -2100F, Japan) was used to confirm the morphology and dimensions of AgNWs and GO films. The topography, morphology and 2D and 3D images for the AgNWs and composite films were investigated by scanning electron microscope (JEOL- IT200, Japan) at an acceleration voltage of 25 kV and atomic force microscopy. SIGNATONE four point-probe was used to measure and calculate the sheet resistance (R_S_) and resistivity of thin films and materials. By moving the lever, allowing the 4 probes to touch the substrate, and applying a current of 4.5 mA, the measured voltage is recorded using a Keithley meter. R_S_ values were estimated using the following equation.

1 $$\text{R}_\text{s} \left( {\frac{\Omega }{\square }} \right) = \frac{\text{Resistivity}}{\text{Thickness}} = \frac{4.53 \times \Delta \text{V}}{\text{I}}$$

where ΔV is the voltage measured by Keithley (mV) and I is the current applied to the probes (mA).

## Results and discussion

### Characterization of prepared AgNWs, GO, and rGO.

#### Optical properties of prepared materials

**Figure 2:**
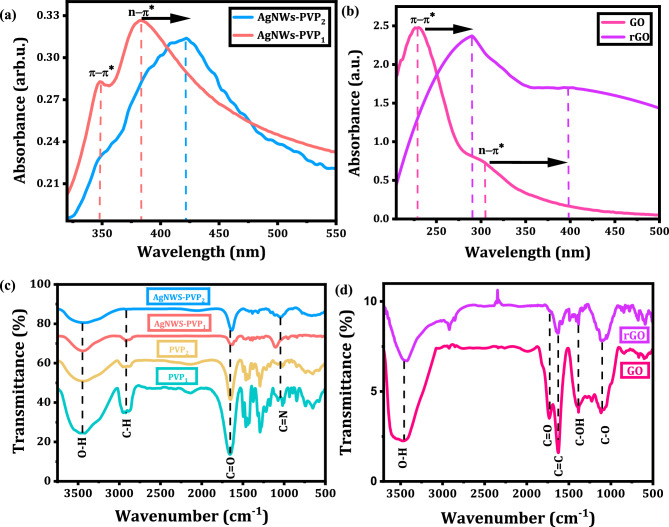
(**a**, **b**) UV–Vis absorption spectra of AgNWs samples with PVP_1_ of Mw = 40, 000 and PVP_2_ of Mw = 1300, 000, GO, and rGO and (**c**, **d**) FTIR absorption spectra of AgNWs samples with PVP_1_ and PVP_2_, pure PVP_1_, pure PVP_2_, GO and rGO.

Figure 2 describes the optical properties of prepared AgNWs, GO, and rGO. The effect of PVP M_W_ on absorbance spectra of AgNWs is illustrated in Fig. 2a. For AgNWs prepared with PVP_1_, the peak located at ~ 348 nm is ascribed to the quadrupole resonance excitation (π–π*) and the narrow peak at ~ 385 nm is corresponded to transverse plasmonic resonance mode (n–π*). This confirms the formation of high yield and pure AgNWs in the sample. However, the red shift in the peak assigned to ≈ 400 nm in AgNWs prepared by PVP_2_ indicates the formation of Ag nanoparticles (AgNPs)^18–20^. At the same time, the broad peak at 400 nm indicates more diverse morphologies of the Ag nanostructures^19–22^.

As shown in Fig. 2b the absorption spectrum of GO solution has two peaks and the main absorption peak at 230 nm is attributed to π–π* transitions of C=C in amorphous carbon system and a broad shoulder with center at ∼ 300 nm that is commonly assigned to n–π* transitions in C=O^23,24^. On the other hand, the peak of the π–π* transition is significantly shifted to 265 nm for rGO, indicating that some groups on the GO surface are reduced and the conjugated structure is restored^24,25^.

Figure 2c represents infrared spectrum of pure PVPs and AgNWs synthesized by PVP_1_ and PVP_2_. The FTIR spectra of pure PVPs show that the peaks concentrated around 1660 cm^−1^ and 2950 cm^−1^ correspond to the vibration of the functional groups –C=O and –C–H, respectively. The absorption peak at 1290 cm^−1^ is also due to the vibration of the functional group –C–N–. Also, the vibration peak associated with the O–H functional group is located at 3445 cm^−1^. The strength of the coordination interaction between Ag^+^ ions and PVP can be estimated in terms of the magnitude of band shifts. It is noticed that the sequence of the strength of the coordination interaction between Ag^+^ ions and PVPs occurs as follows: PVP_2_ M_W_ of 1300,000 > PVP_1_ M_W_ of 40,000, and this confirms that with higher M_W_ of PVP, the production of limited growth of AgNWs occurs and different morphologies of silver nanocrystals are formed^20^.

Figure 2d displays FTIR spectra of GO and rGO powders, there is a broad peak at 3413 cm^−1^ assigned to O–H stretching in hydroxyl, carboxyl groups, and water molecules. The peak around 1620 cm^−1^ is attributed to C=C stretches from unoxidized graphitic domain and transmittance band at 1725 cm^−1^ of C=O stretch of carboxyl group and ketones which are adjacent to benzene rings is observed. In addition, the transmittance band at 1070 cm^−1^ corresponds to the edge phenols and the high-intensity sharp peak at 1380 cm^−1^ of C–OH stretch of alcohol group is noted. The presence of 1210 and 1100 cm^−1^ bands is ascribed to vibration of the epoxy groups and peroxides or five-membered ring lactols, receptively^26–29^. All of these oxidation groups alter the van der Waals interactions among GO layer^30^. Although rGO spectrum has the same features of GO, the intensities of these peaks are declined indicating the success of decomposition of these groups during reduction process of GO by hydrazine and heat treatment^31,32^.

**Figure 3:**
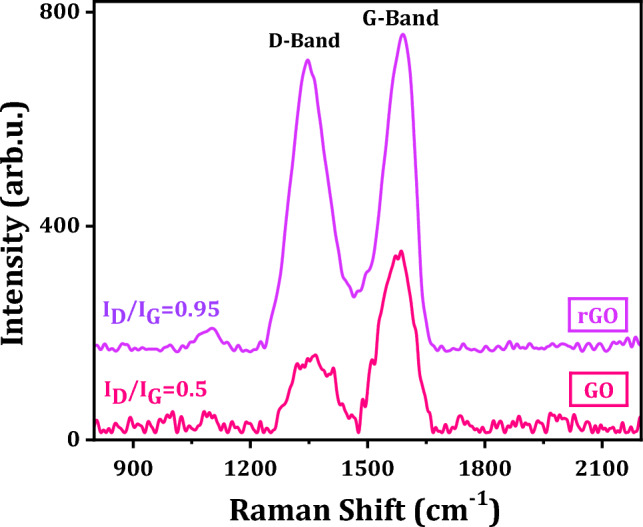
Raman spectra of GO and rGO powders.

**Figure 4:**
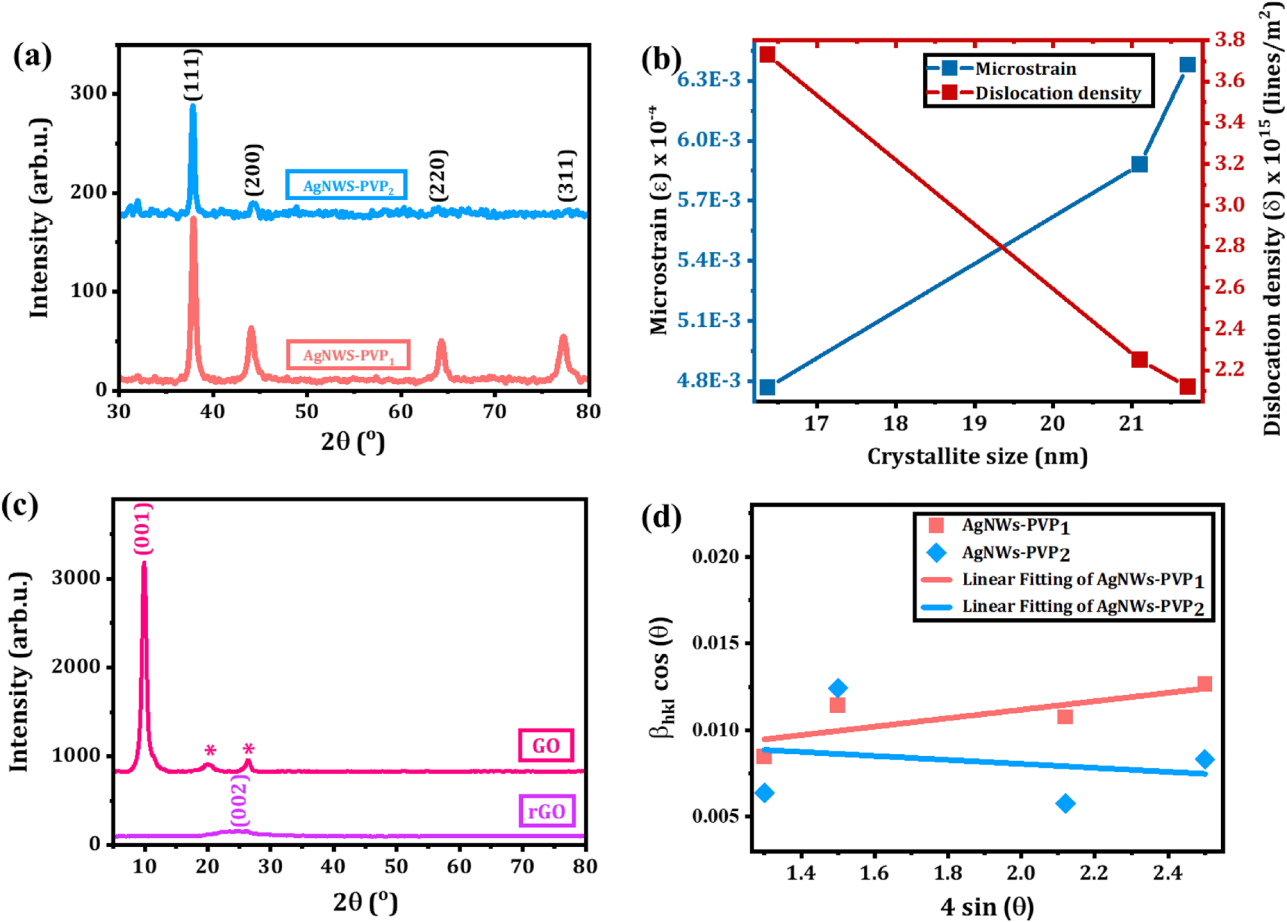
(**a**) XRD patterns and indexing of AgNWs samples with PVP_1_ and PVP_2_, (**b**) crystallite size of AgNWs versus microstrain and dislocation density, (**c**) W–H plot extracted from XRD patterns of AgNWs with PVP_1_ and PVP_2_ samples, and (**d**) XRD patterns and indexing GO and rGO.

Raman spectroscopy is a simple and powerful characterization tool to determine structure, type of graphitic structures and number of graphene layers. Here in Fig. 3, Raman spectrum of GO exhibits two characteristic peaks at 1352 and 1594 cm^−1^. The sharp order G-band at 1594 cm^−1^ results in the first order scattering of E_2g_ phonon characteristic of sp^2^ hybridization of C–C bonds (i.e., in-plane vibrational mode of C–C bonds). On the other hand, the weak D-band at 1352 cm^−1^ originates from the breathing oscillation of k-point phonons with A_1g_ symmetry. The presence of D-band indicates defects and disorders in GO structure due to the oxidation of the carbon bonds^17^. The order and disorder crystal structures of rGO have been intensely observed by the intensity ratio I_D_/I_G_ between D and G band as displayed in Fig. 4. An observation is that the higher disorder rGO leads to a higher relative intensity D band than in GO. It is found that I_D_/I_G_ ratios are 0.45 and 0.95 for GO and rGO, respectively, and this indicates a low degree of defects of GO and the successful thermal and chemical reduction to rGO^33^.

#### Structural and morphological properties of GO, rGO and AgNWs

The crystalline structure of the AgNWs, GO, and rGO are identified through XRD analysis as shown in Fig. 4. The AgNWs samples with different M_W_s of PVP exhibit typical XRD patterns of center cubic structure of silver metal as depicted in Fig. 4b. The four characteristic Bragg’s reflection peaks of AgNWs-PVP_1_ sample are indexed as (111), (200), (220) and (311) with the highest intensity peak at 2θ of 37.9° ascribed to the (111) plane as illustrated in Fig. 4a (JCPDS, Card No. 04-0783)^34^. AgNWs-PVP_2_ pattern suffers from disappearance of the (220) and (311) reflection peaks and all the collected data from them are ambiguous.

The standard intensity ratio between (111)/(200) reflection planes and the (111)/(220) reflection planes are 2.5 and 4, respectively, which demonstrate a preferential growth of the AgNWs in (111) plane (i.e. both ends of silver nanowires). However, by using higher M_W_s of PVP, the intensity ratio decreases, since PVP is coated on the (111) reflection plane and limits their natural growth (i.e. (111) reflection plane is not the preferred orientation of growth) as listed in Table 1^11,35^. Also, the sharpness of Bragg’s reflection planes indicates the crystalline nature of the AgNWs samples. To quantify the crystalline nature of AgNWs samples, the degree of crystallinity was evaluated via the percent relative crystallinity (X_c_) by using Eq. (2)^36^:

2 $${\text{X}}_{{\text{c}}} = \left( {\frac{{{\text{A}}_{{\text{c}}} }}{{{\text{A}}_{{\text{c}}} + {\text{A}}_{{\text{a}}} }}} \right) \times 100\%$$

where A_c_ and A_a_ are the areas of crystalline and amorphous peaks, respectively.

**Table 1:** Crystallographic parameters were extracted from XRD pattern of the AgNWs, GO, and rGO^35,42^.

Sample	No	hkl	(111/200)	(111/220)	2θ_XRD_ (^o^)	2θ_(JCPDS)_ (^o^)	d_hkl(XRD)_ (nm)	d_hkl(JCPDS)_ (nm)	% of expansion in d_hkl_	a (nm)	V (nm^3^)	FWHM (Radian)	D (nm)	Microstrain (ε)	Microstrain (ε) (W–H plot)	Dislocation density [lines/m^2^]
AgNWs-PVP_1_	1	(111)	2.7	3.4	37.929	38.117	0.2370	0.2359	0.47	0.4105	0.0697	0.0089	16.37	4.8 × 10^–3^	2.4 × 10^–3^	3.73 × 10^15^
	2	(200)			44.076	44.279	0.2053	0.2044	0.44	0.4106	0.0692	0.0123	12.12			
	3	(220)			64.311	64.428	0.1447	0.1445	0.14	0.4094	0.0686	0.0125	12.90			
	4	(311)			77.283	77.475	0.1234	0.1231	0.24	0.4091	0.0685	0.0156	10.96			
	D_Average_ = 13.09 ± 2.02 nm (Debye–Scherrer’s equation)								D_Average_ = 22.01 nm (W–H plot)							
AgNWs-PVP_2_	1	(111)	1.3	1.3	37.866	38.117	0.2374	0.2359	0.64	0.4112	0.0695	0.0067	21.71	6.4 × 10^–3^	− 1.15 × 10^–3^	2.12 × 10^15^
	2	(200)			44.322	44.279	0.2042	0.2044	− 0.09	0.4084	0.0681	0.0134	11.16			
	3	(220)			63.969	64.428	0.1454	0.1445	0.62	0.4113	0.0696	0.0067	24.04			
	4	(311)			77.653	77.475	0.1229	0.1231	− 0.02	0.4075	0.0677	0.0103	16.66			
	D_Average_ = 18.39 ± 4.96 nm (Debye–Scherrer’s equation)								D_Average_ = 13.38 nm (W–H plot)							
GO	1	(100)			9.913		0.8916					0.8270	9.64			
rGO	1	(220)			24.270		0.3664					0.1131	1.25			

The percentage of crystallinity is calculated to be 92.7%, and 47.3% for the AgNWs samples with PVP of Mw 40,000, and 1300,000, respectively. It is observed that the higher M_W_ PVP affects badly (limitation of preferred growth in (111) reflection plane) on the crystallinity of the AgNWs samples. For a deeper analysis of the AgNWs samples versus the Mw of PVP by using XRD pattern, the main crystallite size (D) and average crystallite size (D_Average_) is estimated via Debye–Scherrer’s equation (Eq. 3)^37^:

3 $${\text{D}} = \frac{{{\text{k}}\uplambda }}{{\upbeta _{{{\text{hkl}}}} \left( {2\uptheta } \right)\cos \left(\uptheta \right)}}$$

where β_hkl_ (2θ) is full width at half maximum (FWHM), hkl are the miller indices of the crystal lattice, k is a constant known as a shape factor = 0.9, λ is the wavelength of X-ray radiation, and 2θ is Bragg’s diffraction angle.

As listed in Table 1, the mean crystallite size of the AgNWs samples of the characteristic peak (111) increases as the Mw of the PVP increases. To qualify the crystal structure and the unit cell formation of the AgNWs samples, the interlayer spacings (d-spacings, d_hkl_) between AgNWs planes were detected by applying Bragg’s Law (Eq. 4) and listed in Table 1.

4 $${\text{n}}\uplambda = 2 {\text{d}}_{{\text{hkl }}} \sin \left(\uptheta \right)$$

where n is an integer number and d_hkl_ is the interlayer spacing between atoms.

The d-spacing of the characteristic peak (111) were found to be a bit higher than the standard values, as listed in Table 1^34^. Also, the lattice constant (a) of the AgNWs cubic unit cell was also calculated via Eq. (5)^38^.

5 $$\frac{1}{{{\text{d}}_{{{\text{hkl}}}}^{2} }} = \left[ {\frac{{{\text{h}}^{{2{ }}} + {\text{k}}^{2} + {\text{l}}^{2} }}{{{\text{a}}^{2} }}} \right]$$

where a is the lattice constant for the cubic unit cell.

The lattice constant (a) of (111) plane for the AgNWs samples is found to be slightly higher than the standard database (a = 0.4086 nm)^34^, as listed in Table 1. Notably, both the d-spacing and the lattice constant of the AgNWs samples increased by increasing the Mw of the PVP. Hence, it implies an increase in the distortion of the unit cell formation by increasing the Mw of the PVP, which is previously confirmed by the reduction in the percentage of crystallinity. Consequently, microstrain fluctuations and dislocations are formed in the crystal structure of the AgNWs samples.

The microstrain fluctuations (ɛ) in crystal structure are extracted from the lattice constants equation to be:

6 $$\upvarepsilon = \frac{{a - a_{o} }}{{a_{o} }}$$

where a is the measured lattice constant for the AgNWs sample and a_o_ is the standard lattice constant for silver metal.

As shown in Fig. 4b and listed in Table 1, AgNWs samples exhibit tensile macrostrain fluctuations, and their values increase as the Mw of the PVP and crystallite size increases. The tensile microstarin is attributed to the lattice mismatch of the AgNWs due to the increase in the Mw of PVP. Hence, it explains the previously noticed % of expansion in d_hkl_ and lattice constant values as compared with the standard database JCPDs. Also, it elucidates the shift of diffraction angles toward lower values as compared with standard. Subsequently, dislocations between the AgNWs atomic planes were induced as a reflection of the lattice mismatch and macrostrain fluctuations. The dislocations density (δ) in were calculated from 1/D^2^ (D is the main crystallite size)^39^, as shown in Fig. 4b and listed in Table 1. It is obvious that by increasing the Mw of the PVP in the AgNWs samples, the particle size increases, and the dislocation density decreases. Interestingly, the average crystallite size (D) and the microstrain fluctuations (ε) can be also calculated by applying Williamson–Hall (W–H) expression (Eq. 6)^40^:

7 $$\upbeta _{{{\text{hkl}}}} \left( {2\uptheta } \right) \cos \left(\uptheta \right) = \frac{{{\text{k}}\uplambda }}{{\text{D}}} + 4 \upvarepsilon \,\sin \left(\uptheta \right)$$

As shown in Fig. 4c, the average crystallite size and the microstrain fluctuations were extracted from intercept and the slope of the W–H plot, respectively and listed in Table 1. Notably, the average crystallite size and microstrain fluctuations that were calculated from the W–H differ than those from Debye–Scherrer’s method. The discrepancy in the values from both methods is attributed to existence of diverse geometries along with microstrain in d_hkl_.

Figure 4d illustrates the XRD patterns of GO and rGO. XRD patten of GO exhibits sharp and characteristic reflection plane (100) at 2θ of 9.91° with a corresponding d-spacing of 0.8916 nm, which is a good consistency with literature^41^. The high value of the d-spacing confirmed the intercalation of the oxygen atoms, water molecules, and other structural defects between graphitic layers in the GO, which bound to the graphite planar surface^42,43^. Also, two shoulder peaks at 2θ of 20.05° and 26.42° are appeared in the XRD pattern of the GO, which are attributed to a fraction of oxygen atoms was not fully intercalated into GO^10,11^. On the other hand, rGO pattern is displayed with broad characteristic reflection peak (220) at 2θ of 24.27° and d-spacing of 0.3664 nm, which is a good consistency with literature^41^.

**Figure 5:**
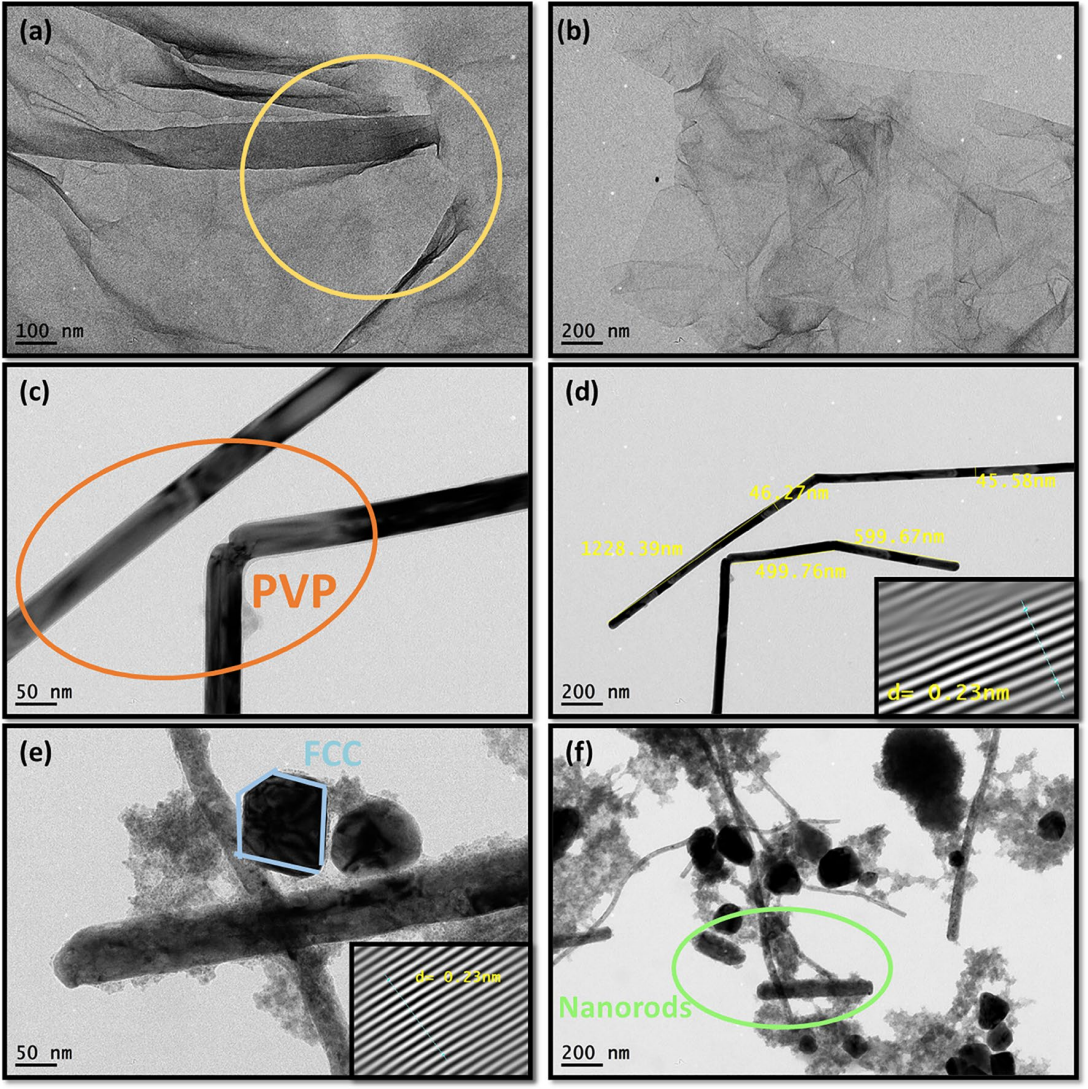
TEM and HRTEM micrographs of: (**a**, **b**) GO with two different magnifications, (**c**) AgNWs/PVP_1_ solution without treatment with orange circle demonstrate excess of PVP, (**d**) width and length measurements on AgNWs/PVP_1_ sample to calculate its aspect ratio and the inset depicts d-spacing, (**e**) AgNWs sample/PVP_2_ with face centered cubic (FCC) structure and the inset depicts d-spacing, and (**f**) AgNWs/PVP_2_ sample shows nonuniformity and different Ag nanostructures.

Morphological property of GO and AgNWs is illustrated and confirmed by HRTEM micrographs as displayed in Fig. 5. It can be seen from Fig. 5a,b that GO exhibits a typical translucent sheet structure with curls and wrinkles in the middle portion inside yellow circle. GO HRTEM micrographs present diverse layers and lateral dimensions with folded structure due to a deformation during peeling and the presence of attraction forces of functional groups (–OH, –COOH, C=O and C–O–C)^44,45^.

The detailed morphology of a long and thin AgNWs prepared by PVP_1_ is shown in Fig. 5c, AgNWs/PVP_1_ exhibits homogeneity and sharp end face that comprised (111) planes^35,46^. Furthermore, a thin and lighter contrast coating layer of PVP is clearly wrapped onto the surface of AgNWs so post-treatment step is crucial to remove excess PVP and improve the junction among wires^47^. Obviously, Fig. 5d illustrates high aspect ratio with an average uniform diameter ≈ 46 nm and length ≈ 1.2 µm, and the lattice spacing of 0.23 nm which is in agreement with the d-spacing value of the (111) plane of face centered cubic (FCC) in XRD patterns of AgNWs/PVP_1_.

Figure 5e describes the lattice spacing of 0.23 nm and FCC structure which are characteristic to formation of AgNWs prepared by PVP_2_ solution. However, there are more diverse morphologies of Ag NPs, Ag nanorods and lower yield of thick AgNWs in sample prepared by PVP_2_ as shown in Fig. 5f. In conclude, the lower the M_W_ of PVP the more uniform formation of AgNWs^48^. In addition, to fabricate high transparent conductive electrodes based on pure AgNWs, samples of AgNWs synthesized by PVP_1_ are recommended to be used.

### Characterization of fabricated TCEs

#### Morphological and topographical properties of TCEs

**Figure 6:**
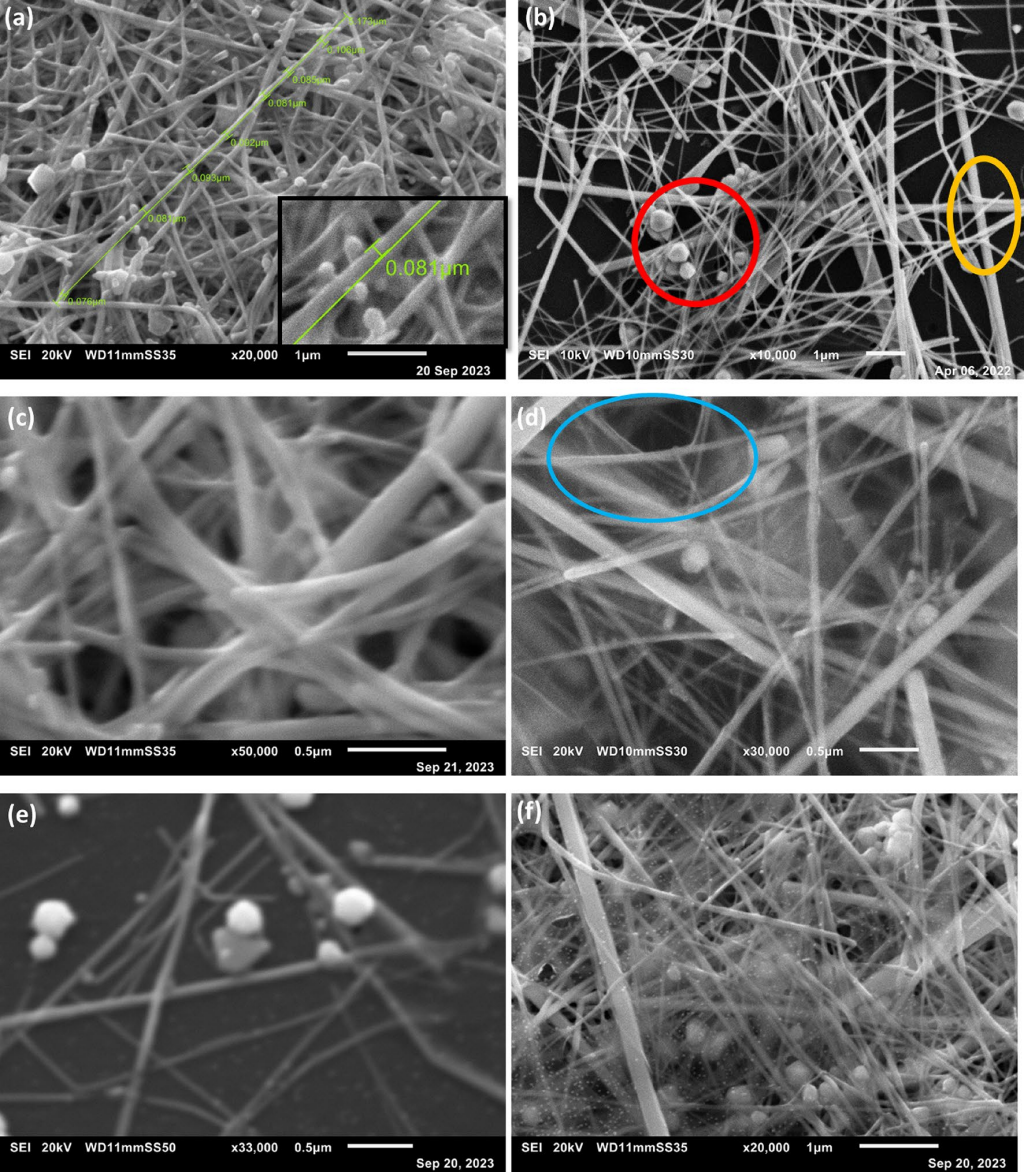
SEM micrographs of: (**a**) Annealed AgNWs electrode show sea of AgNWs network with a uniform diameter of 81 nm (**b**) Pentagonal, spherical particles and bright spots of welded nanowires junctions circled by the red and orange circles appeared from top view of AgNWs electrode, (**c**, **d**) 30° tilting angle and top view of of AgNWs/ PEDOT:PSS composite electrode at different magnifications, and (**e**, **f**) AgNWs/rGO electrode at different magnifications with fully covered layer of rGO above AgNWs electrode.

SEM micrographs are obtained for AgNWs, AgNWs/ PEDOT:PSS, and AgNWs/rGO electrodes from top view in the right side and 30° tilting angle view in the left side, as shown in Fig. 6. SEM micrographs of annealed AgNWs electrode at 150 °C for 10 min. in air are illustrated in Fig. 6a. Apparently, long and one-dimensional shapes with uniform diameter of 81 nm and average length of 12 µm are observed indicating the high aspect ratios of synthesized AgNWs. However, some pentagonal and spherical structures of AgNWs are noticed and distributed in the sea of AgNWs network indicating that AgNWs grew with a pentagonal cross-section as shown in Fig. 6b. Additionally, it is noted that there are bright spots as shown in yellow color circle among AgNWs crosslinks indicating good connectivity and conductivity of AgNWs^49^.

Furthermore, AgNWs/PEDOT:PSS composite electrodes with a thin and transparent coated layer above AgNWs are observed in Fig. 6c,d. On the other hand, there are some areas with dark shadows indicating non fully coverage of AgNWs. In contrast, AgNWs/rGO composite electrodes display high coverage, homogeneity with low roughness in Fig. 6e,f. In high magnification, rGO layer improves the connection among AgNWs network because the GO film wrapped around the AgNWs, which improved the inter-nanowire junction and achieves the composite goal^50^.

**Figure 7:**
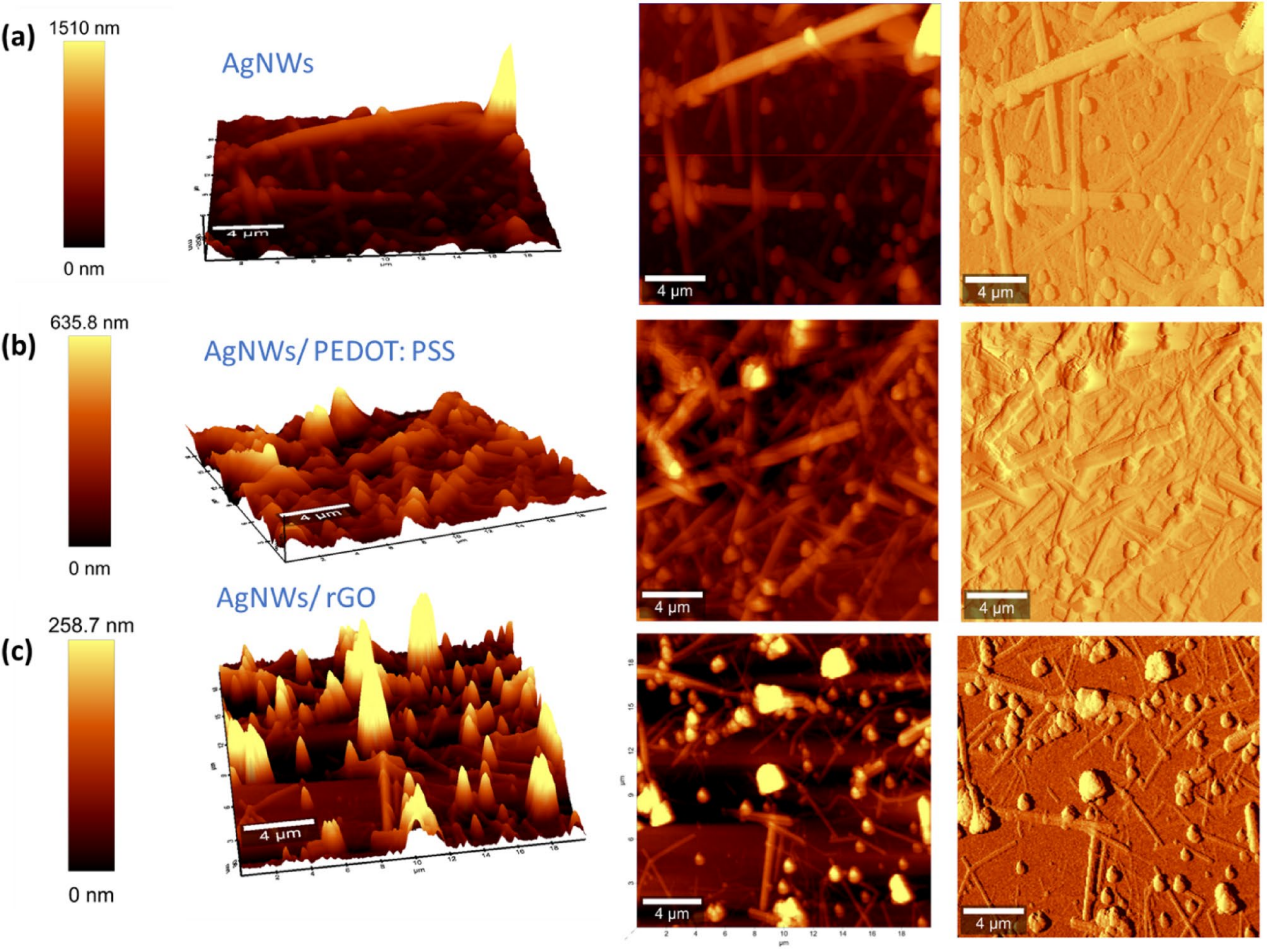
2D and 3D topographical AFM images of different TCEs on a glass substrate: (**a**) AgNWs film, (**b**) AgNWs/PEDOT:PSS composite film, and (**c**) AgNWs/rGO composite film.

The surface topography, roughness and 3D images of AgNWs, AgNWs/PEDOT:PSS, and AgNWs/rGO electrodes are studied and illustrated using AFM as shown in Fig. 7. The topographic image of AgNWs electrode has a rough surface with a max height of 1510 nm as shown in Fig. 7a. This high roughness value indicates the abnormal thickness of multilayered AgNWs electrode and there are imperfections among the nanowires. The presence of dark areas is associated with network discontinuities due to an incomplete surface coverage^16^. However, the high coverage is achieved by fabricating AgNWs/PEDOT:PSS composite electrode on glass substrate as displayed in Fig. 7b. It is observed that the roughness value of AgNWs/PEDOT:PSS composite electrode declines to 635.8 nm. AFM images of AgNWs/rGO composite film illustrated in Fig. 7c have been smoothened to 258.7 nm attributed to embed of AgNWs in rGO matrix. The composites roughness is considerably reduced relative to the surface roughness of the bare AgNWs mesh^51–53^.

#### Surface energy of TCEs

**Figure 8:**
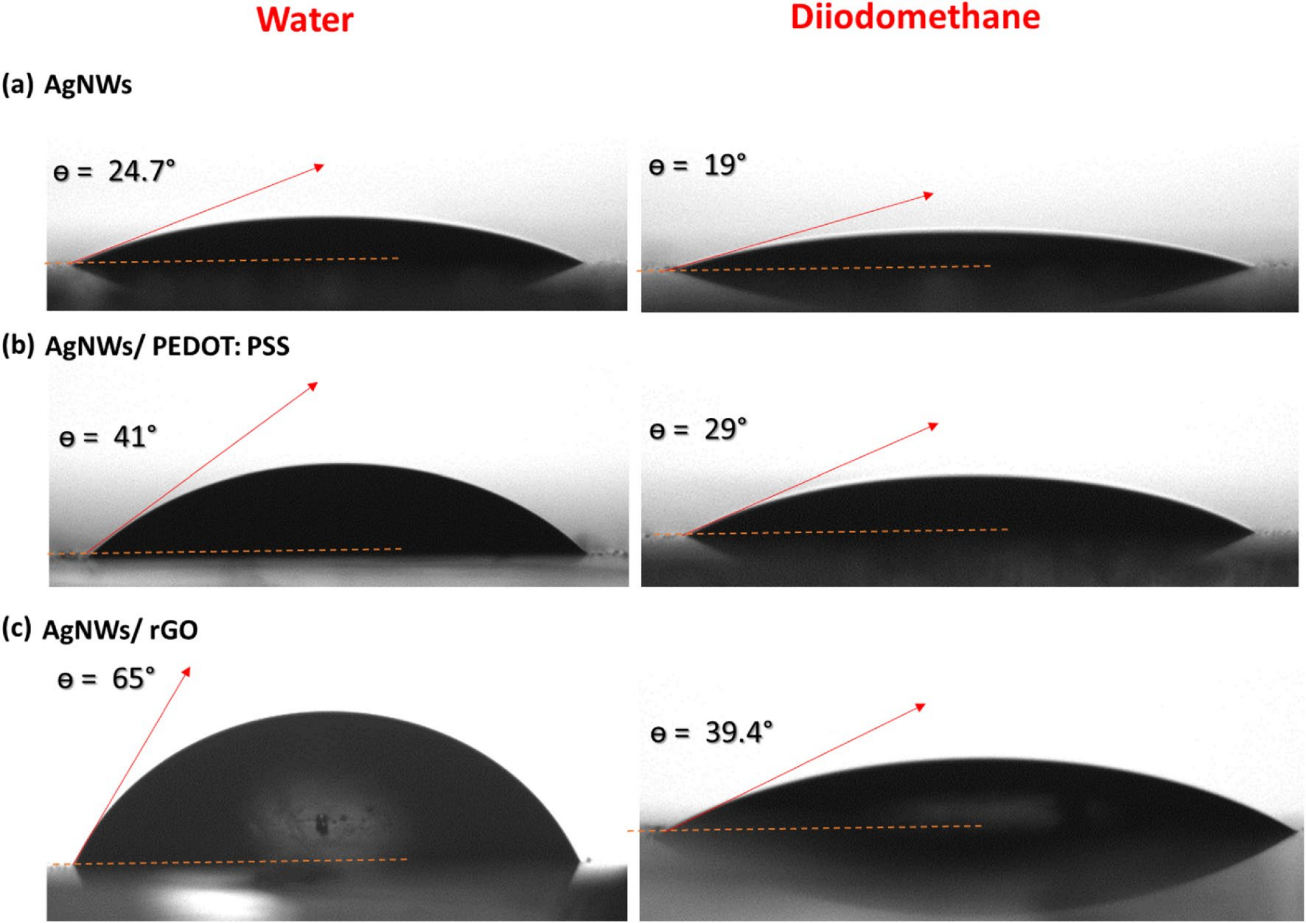
Contact angle measurements for the surface of (**a**) AgNWs, (**b**) AgNWs/PEDOT:PSS composite, and (**c**) AgNWs/rGO composite electrodes using a drop of water in the left side and a drop of diiodomethane in the right side.

In order to form a transparent conductive film with composite materials, AgNWs surface has to have high wettability. As shown in Fig. 8a the contact angle between the tangent of water and AgNWs surface is found to be 24.7°. The hydrophilic behavior of AgNWs film is due to the high surface roughness and consequently liquid penetrates the grooves of AgNWs as observed in AFM images. On the other hand, 41° and 65° contact angles are obtained when a drop of water is placed on composite electrodes of AgNWs/PEDOT:PSS, and AgNWs/rGO, respectively as presented in Fig. 8b,c. Composite electrodes with smoother surfaces produce higher water contact angles.

Contact angles of diiodomethane are measured to evaluate the surface energy values of TCEs. Surface energy is calculated from a set of liquid/solid contact angles. There are several theories for converting contact angle data into solid surface energy values^54^. Fowkes theory is based on primary equations which describe interactions between solid surfaces and liquids. These Eqs. (8–10) are as follows:

8 $$\left( {{\upsigma }_{L}^{D} } \right)^{\frac{1}{2}} \left( {{\upsigma }_{S}^{D} } \right)^{\frac{1}{2}} + \left( {{\upsigma }_{L}^{P} } \right)^{\frac{1}{2}} \left( {{\upsigma }_{S}^{P} } \right)^{\frac{1}{2}} = \frac{{{\upsigma }_{L} (\cos \theta + 1)}}{2}$$

9 $${\upsigma }_{L} = {\upsigma }_{L}^{P} + {\upsigma }_{L}^{D}$$

10 $${\upsigma }_{S} = {\upsigma }_{S}^{P} + {\upsigma }_{S}^{D}$$

where $\upsigma _{{\text{L}}}^{{\text{D}}}$ is dispersive component of the surface tension of liquid, $\upsigma _{{\text{L}}}^{{\text{P}}}$ polar component of the surface tension of liquid, $\upsigma _{{\text{S}}}^{{\text{D}}}$ is dispersive component of the surface energy of the solid, and $\upsigma _{{\text{S}}}^{{\text{P}}}$ is polar component of the surface energy of the solid, and θ is the contact angle between solid and liquid. σ_L_ is overall surface tension of the wetting liquid, and σ_S_ is overall surface energy of the solid.

Fowkes theory is applied using contact angle data from only two liquids and the recommended liquids are diiodomethane and water. To determine solid surface energy, the contact angle between solid surface and a drop of diiodomethane which has only dispersive component is measured ($\upsigma _{{\text{L}}}^{{\text{P}}}$  = 0, and $\upsigma _{{\text{L}}}^{{\text{D}}}$ = σ_L_). In this case, the Eq. (8) reduces to:

11 $${\upsigma }_{S}^{D} = \frac{{{\upsigma }_{L} (\cos \theta + 1)^{2} }}{4}$$

By substitution in the above equations by the value of σ_L_ for diiodomethane (50.8 mN/m), and for water σ_L_^D^ (26.4 mN/m) and σ_L_^P^ (46.4 mN/m). The overall solid surface energy values for AgNWs, AgNWs/PEDOT:PSS, and AgNWs/rGO electrodes are 72.75, 63.44, and 47.95 mN/m, respectively. The surface energies of the composite electrodes are decreased with covering AgNWs with smother layer^55,56^. In addition, AgNWs/rGO composite electrode with the lowest value of surface energy confirms the formation of large nano islands and thus lower surface diffusion process.

#### Electro-optical properties of fabricated TCEs

**Figure 9:**
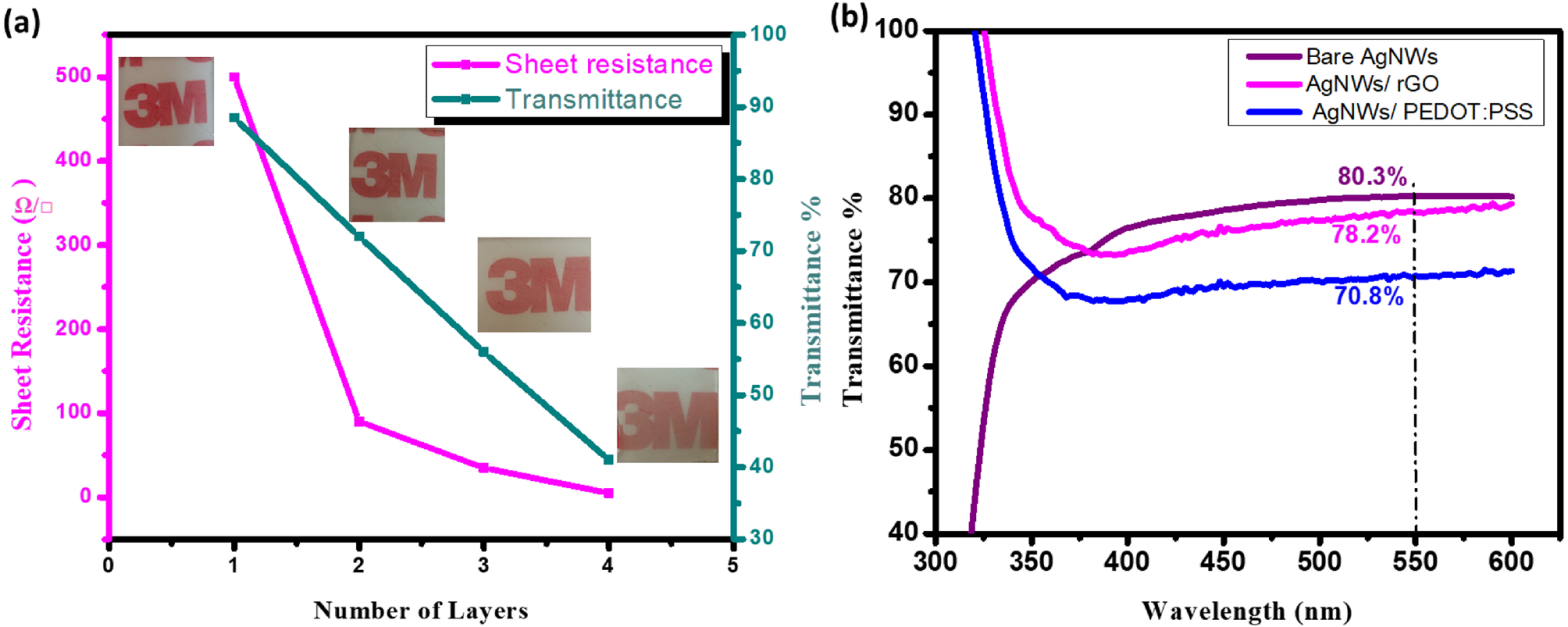
(**a**) Relation between transmittance percentage and sheet resistance of AgNWs electrode as a function of number of deposited layers (**b**) transmittance spectra in the visible region for pure AgNWs, AgNWs/rGO composite, and AgNWs/PEDOT:PSS composite electrodes.

The electro-optical properties are measured and discussed on AgNWs, AgNWs/rGO, and AgNWs/PEDOT:PSS electrodes after a thermal annealing step as it significantly decrease the resistance without having an impact on their transmittance^57^. The effect of number of layers deposited by AgNWs, and composite electrodes on sheet resistance and transmittance values has been recorded in Table 2. It is noted that there is an inverse relationship between number of layers and both sheet resistance and transmittance of AgNWs electrodes as plotted in Fig. 9a, since increasing number of deposited layers means higher yields of AgNWs that formed and crosslinked together which obviously increase the electrons mobility and consequently reduce the sheet resistance. However, the transmittance values have significantly decreased due to higher diffusion caused by grouping of Ag nanostructures on the glass substrates^10^.

**Table 2:** Transmittance and sheet resistance measurements as a function of number of layers of TCEs.

TCEs	Number of layers	Sheet Resistance (Ω/□)	Transmittance (%)
Bare AgNWs	1	85	80.3
	2	67	72
	3	23	56.5
	4	5	41
AgNWs/PEDOT:PSS	1	28	70.8
	1		
AgNWs/rGO	1	27	78.2
	1

On the other hand, the composite electrodes AgNWs/rGO and AgNWs/PEDOT:PSS have significant effect on the reduction of sheet resistance, while a small change in transmittance values is observed in Fig. 9b. Embedding AgNWs in PEDOT:PSS or rGO upper layers to form large hybrid areas assists in enhancing the conductivity and lowering the transparency of C-TCEs^58^. Notably, AgNWs/rGO composite electrode has won the trade-off between optical and electrical properties confirming its ability to perform well in optoelectronic applications.

As listed in Table 3. The presented study is compared with the latest in literature. However, slight changes in values are observed in favor of literature, which is attributed to the differences in the preparation method. It is worth being mentioning that the presented study offers simple, fast, and cost-effective synthesis technique for the synthesis of AgNWs with a comparable result as compared with literature. All these minor changes will be greatly considered in our future work.

**Table 3:** Comparison between latest literature and our work.

TCEs	Sheet resistance (Ω/□)	Transmittance (%)	Materials and method	References
Our work				
Bare AgNWs	85	80.3	AgNWs was prepared by hydrothermal and GO by modified hummers method	
AgNWs/PEDOT:PSS	28	70.8		
AgNWs/rGO	27	78.2		
Examples from literature				
AgNWs	43–33	87–92.7	Synthesized by polyol method, Purchased and grid-patterned	^59,60^
AgNWs/PEDOT:PSS	35–21.2	88–80.5	Synthesized by polyol method and purchased	^59,61^
AgNWs/rGO	25–20	87.6–84.5	Synthesized by polyol method and	^62,63^

## Conclusions

AgNWs were synthesized by a simple hydrothermal method using two M_W_s of PVP. A high aspect ratio and high yield with uniform diameter of AgNWs were obtained using PVP of 40,000. Additionally, the higher M_W_ of PVP of 1300,000 led to a larger and non-uniform morphologies of Ag nanostructures, and lower percentage of crystallinity with diversity of Ag nanostructures which consequently reduced the connection between wires and the sheet resistance of the AgNWs film. The optical, structural, morphological, and topographical properties of prepared materials and C-TCEs are investigated in this article. Covering the AgNWs surface with protective layer from rGO obviously enhanced the AgNWs junction and sheet resistance and showed a little effect on the transmittance. The optimum transparent conductive electrodes possessed high transparency of (78.2%) and low sheet resistance of 27 Ω/□. The electrical properties of C-TCEs exhibited high stability when sheet resistance and transmittance were measured after one month of fabrication process without applying any mechanical forces.

## Data Availability

The authors confirm that the data supporting the findings of this study are available and can be accessed within the article.
